# Mothers' education but not fathers' education, household assets or land ownership is the best predictor of child health inequalities in rural Uganda

**DOI:** 10.1186/1475-9276-3-9

**Published:** 2004-10-13

**Authors:** Henry Wamani, Thorkild Tylleskär, Anne Nordrehaug Åstrøm, James K Tumwine, Stefan Peterson

**Affiliations:** 1 Centre for International Health, University of Bergen, Armauer Hansen Building, N-5021 Bergen, Norway; 2 Ministry of Health, P.O Box 7272, Kampala, Uganda; 3 Department of Paediatrics and Child Health, Makerere University Medical School, P.O Box 7072, Kampala, Uganda; 4 Division of International Health (IHCAR), Karolinska Institute, Norrbacka, S-17176 Stockholm, Sweden

**Keywords:** Child health, inequalities, stunting, socio-economic, targeting, mother education, Uganda

## Abstract

**Background:**

Health and nutrition inequality is a result of a complex web of factors that include socio-economic inequalities. Various socio-economic indicators exist however some do not accurately predict inequalities in children. Others are not intervention feasible.

**Objective:**

To examine the association of four socio-economic indicators namely: mothers' education, fathers' education, household asset index, and land ownership with growth stunting, which is used as a proxy for health and nutrition inequalities among infants and young children.

**Methods:**

This was a cross-sectional survey conducted in the rural district of Hoima, Uganda. Two-stage cluster sampling design was used to obtain 720 child/mother pairs. Information on indicators of household socio-economic status and child anthropometry was gathered by administering a structured questionnaire to mothers in their home settings. Regression modelling was used to determine the association of socio-economic indicators with stunting.

**Results:**

One hundred seventy two (25%) of the studied children were stunted, of which 105 (61%) were boys (p < 0.001). Bivariate analysis indicated a higher prevalence of stunting among children of: non-educated mothers compared to mothers educated above primary school (odds ratio (OR) 2.5, 95% confidence interval (CI) 1.4–4.4); non-educated fathers compared to fathers educated above secondary school (OR 1.7, 95% CI 0.8–3.5); households belonging in the "poorest" quintile for the asset index compared to the "least poor" quintile (OR 2.1, 95% CI 1.2–3.7); Land ownership exhibited no differentials with stunting. Simultaneously adjusting all socio-economic indicators in conditional regression analysis left mothers' education as the only independent predictor of stunting with children of non-educated mothers significantly more likely to be stunted compared to those of mothers educated above primary school (OR 2.1, 95% CI 1.1–3.9). More boys than girls were significantly stunted in poorer than wealthier socio-economic strata.

**Conclusions:**

Of four socio-economic indicators, mothers' education is the best predictor for health and nutrition inequalities among infants and young children in rural Uganda. This suggests a need for appropriate formal education of the girl child aimed at promoting child health and nutrition. The finding that boys are adversely affected by poverty more than their female counterparts corroborates evidence from previous studies.

## Background

Health outcomes are a result of a multi-layered conceptual framework with proximal and distal determinants [1]. Proximal (nearer in time and place to the terminal event) determinants such as feeding practices, injury and disease especially the infectious diseases are mediated through factors prevailing within households or the institutions serving children directly. At the distant level are factors operating through the socio-political and economic dimensions of the environment.

In recent decades, development agencies and governments have emphasized efficacious interventions to improve child survival and healthy development. However, most of these interventions have traditionally addressed proximal determinants with limited effort on distal determinants since many of them lie outside the health sector. Recent calls were made for interventions to address the more distal determinants of health outcomes [2,3]. To minimize inequities in child health and nutrition emphasis is made on correct identification and service delivery to the poor and most vulnerable in society.

To assess inequalities of child health and nutrition, the World Health Organisation recommends use of linear growth retardation (stunting) [4-6]. In the biomedical arena stunting is attributable to a wide range of prenatal and postnatal factors. These include: low birth weight [7-9], inadequate care and stimulation [10], and insufficient nutrition and recurrent infections [11]. Use of stunting avoids the measurement pitfalls like improper case definitions, time, and others usually associated with more traditional measures such as morbidity, mortality, and life expectancy. It also eludes problems associated with using monitory variables such as income or expenditure to compare health inequalities [12,13].

Stunting varies systematically with socio-economic status with the poorest most affected by it. This relationship is observed in a number of studies in the African region [14-18]. Unfortunately, proxies used to construct socio-economic indicator(s) vary. The relative household socio-economic position is sometimes developed by lumping indicators of socio-economic status into one global indicator, or derived from non-interventional feasible indicators. Additionally control for unmeasured effects of each indicator on others is often never done. This is detrimental to development of effective policy since choice of an indicator has interventional implications. This is especially critical in sub-Saharan Africa where evidence suggests that macro-economic growth alone is unlikely to significantly reduce stunting in medium terms [19]. Therefore efforts to address distal determinants of child health inequalities especially through the socio-economic pathway, requires that great care is taken in selecting indicators that are amenable to easy monitoring and feasible interventions [20].

The present study was conducted in a rural setting, in Uganda. It examines the independent influence of four different components of socio-economic status: mothers' education, fathers' education, household asset index, and land ownership on growth stunting, a proxy for health inequalities. Results of this study should provide policy makers and programme managers with useful information in child health and nutrition planning for rural areas.

## Methods

### Study area

The study was carried out in Hoima district, which is located in western Uganda and shares a border with the Democratic Republic of Congo along Lake Albert. The district has a population density of 80 per square kilometre with a total population of approximately 370,000, comprised of mainly peasant farmers of the Banyoro tribe [21]. The district has fertile soils and enjoys a bimodal rainfall pattern ranging from 800 – 1500 mm per annum enabling two harvests a year. Farming activities include cattle rearing, growing tobacco and coffee for cash; and maize, cassava, potatoes, beans and groundnuts for consumption.

### Design and sampling

The present survey utilised a two stage probability proportional to size cluster design [22]. Data was collected with respect to infant- and young child feeding knowledge and practices, anthropometry and indicators for household socio-economic status. The sample was made large enough to estimate a prevalence rate of wasting similar to that assessed at the national level (4.0%), at 95% level of confidence [23], with 2% precision error and an assumed intra-cluster correlation coefficient of 0.11. The national census data [21] was used to estimate the population under-2 years of age in Hoima district (24,000). A sample size of 720 children was calculated based on the formula by Cochran [24], using the SampleXS software. At the first stage of sampling, a total of 72 villages were selected on probability proportional to population size basis. At the second stage, a total of 10 households were systematically sampled from each village selected at the first stage, providing a self-weighting sample with each child/mother pair in the district having an equal probability to be selected into the sample. A household was defined as a group of people living, cooking and eating together. One child under 2 years was enrolled per household after an informed verbal consent of the mother/caregiver. In case a household had two children in the target age group or twins one was selected randomly. Children with major handicap, disability or malformation were excluded from the sample. Problems encountered with sample selection included the existence of newly formed villages hitherto unrecorded in the census register. In one case an old village had been split; one of the "offspring" villages was selected randomly.

### Data Collection

A structured questionnaire constructed in English and translated into Runyoro, the local language, was pilot tested and updated in survey similar settings and administered face-to-face to mothers/caregivers in their home settings. Trained non-medical university students gathered data in September and October 2002. To minimise bias, training of interviewers emphasized proper and random identification of respondents, eligible children, and questionnaire administration. A village leader followed data collectors through the village, and traditional village protocol was observed.

Information about the educational level of parents, household durable assets, materials of the dwelling structure and land ownership was collected as proxy makers of household socio-economic status. Child's age was obtained through birth certificates, health cards, or recall using a calendar of local events while taking precautions to minimize field errors [25]. Recumbent length measurements were taken with specially designed length boards that measured to the nearest 1 mm following standard recommendations [26]. The questionnaire was based on the demographic and health survey (DHS) questions [27] to avoid separate validity and reliability checks of the data collection instrument.

### Measurements

The dependent variable (stunting) was constructed using child length, age and sex, and was defined as length less than minus 2 standard deviations (<-2 SD) from the median of the NCHS/WHO reference population [5].

The education level of fathers and mothers was assessed on scales ranging from (1) never went to school, to (7) college/university. Two categorical variables were constructed yielding four categories for mothers: (1) no formal education, (2) stopped in primary, (3) completed primary (minimum 7 years of school) and (4) stopped above primary. Five categories were yielded for fathers because they were more educated: (1) no formal education, (2) stopped in primary, (3) completed primary, (4) stopped in secondary, and (5) completed secondary 4 or above (minimum 11 years of school).

Household durable assets (cupboard, hurricane lamp, radio, bicycle, fuel, boat, telephone, refrigerator, motorcycle and car) were assessed as (1) available/ in working condition and (2) not available/ not in working condition. Four components of the dwelling structure were assessed including number of rooms, roof – (1) thatched, (2) corrugated iron sheets or tiles; floor – (1) mud, (2) cement; wall – (1) thatched, (2) mud and pole, (3) unburned bricks, (4) burnt bricks built with mud, (5) burnt bricks built with cement and (6) cement/concrete blocks. A household wealth (asset) index was constructed from 15 variables (household durable assets and dwelling structure) using principal components analysis [28]. The index scores were divided into quintiles (1) poorest, to (5) least poor.

Land is usually given special socio-economic status in many subsistence agricultural communities in Uganda [29]. In order to assess for its independent contribution to child well being land ownership was analysed independent of other household assets. It was assessed as a continuous variable using an open question "How much land does your family have for agriculture or any other activity?" A football pitch was used to estimate the size equivalent to one acre. A categorical variable was constructed in terms of; (1) no land at all, (2) one acre, (3) two acres, (4) three acres and (5) four or more acres.

Child age in months was assed as a continuous variable and a categorical variable was constructed yielding four categories: (1) 0–5 months, (2) 6–11 months, (3) 12–17 months, and (4) 18–23 months. Child sex was ordinal (1) male, (2) female.

### Analysis

Data was entered in Epidata software [30], and was analysed in epi info (version 6.04d) and SPSS (version 12.0). Of 720 children recruited, 23 (3%) had missing data on anthropometric measurements or were extreme outliers and were dropped from the analysis of stunting. First, socio-economic indicators (mothers' and fathers' education, household wealth index and land ownership) were evaluated for the extent of shared variation by obtaining bivariate correlation coefficients (Spearman's rho). Second, unadjusted associations of stunting with socio-economic and demographic variables were examined by use of odds ratios. Third, backward conditional logistic regression was done simultaneously controlling for socio-economic and demographic factors. The pattern of stunting with sex across different socio-economic strata was evaluated with chi-square tests and bivariate logistic regressions. Mean age differences between boys and girls were assessed by the student t-test. Tests for trend across categories were performed by treating the categories as continuous variables in the logistic regression analyses. The clustering effect was not controlled for as the large number of primary sampling units (72) in the study minimizes it. The goodness-of-fit for regression models was assessed with Hosmer and Lemeshow test; with the null hypothesis that the model adequately fits the data (significant chi-square p-value implies that the model does not fit).

### Ethics

Approval of the study was granted by Makerere University Faculty of Medicine Ethics and Research committee, the Uganda National Council for Science and Technology and the Regional Committee for Medical Research Ethics, West Norway (REK vest).

## Results

**Figure 1:**
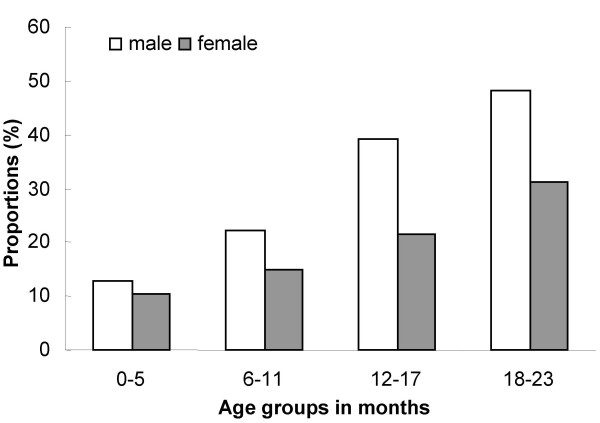
Proportion of stunted children (<-2 SD of NCHS/WHO reference) among 720 children by sex and age group in western Uganda

Of the children studied 366 (51%) were females. The median age (inter-quartile range) was 10 (5–16) months for females and 11 (5–16) months for males respectively. The mean age difference between boys and girls was not statistically significant (p = 0.34). A total of 148 (21%) mothers and 59 (9%) fathers never attained any formal education (Table 1), while only 52 (7%) of mothers were educated up to secondary 4 or above. Ninety percent of mothers were married. One hundred seventy two (25%) of the children in the study were stunted out of which 105 (61%) were boys. The status of stunting ranged from 12% among children aged 0–5 months to 41% in children 18–23 months (Figure 1). There were 21 twins included in the study and 6 of them were stunted. Ninety percent of households owned any land. Median acreage was 2.0 and the mean was 3.6 acres.

**Table 1:** Frequency distribution, unadjusted and conditional multiple logistic regressions of stunting with socio-economic predictors *(coefficients are expressed as odds ratios and p-values for the test of trend are indicated)*

Variables	Total	Stunted^b^	Unadjusted		Adjusted	
	n^a ^(%)	n (%)	OR	95%CI	OR	95%CI
Child age in months						
18–23	150 (22)	61 (41)	5.2	2.9–9.3***	5.1	2.6–9.8***
12–17	189 (27)	56 (30)	3.2	1.8–5.7***	3.0	1.6–5.8**
6–11	195 (28)	36 (19)	1.7	0.9–3.1	1.8	0.9–3.5
0–5	164 (23)	19 (12)	1.0		1.0	
*p-value for trend test*			<*0.001*		<*0.001*	
Child sex is male	354 (49)	105 (31)	1.9	1.3–2.7***	2.0	1.3–3.0**
Mother's education						
None	148 (21)	45 (31)	2.5	1.4–4.4**	2.1	1.1–3.9*
Stopped in primary	282 (40)	80 (30)	2.3	1.4–3.9**	2.1	1.2–3.8*
Completed primary	131 (18)	22 (18)	1.2	0.6–2.2	1.0	0.5–2.0
Above primary	152 (21)	23 (15)	1.0		1.0	
*p-value for trend test*			<*0.001*		*0.03*	
Father's education						
None	59 (9)	17 (30)	1.7	0.8–3.5		
Stopped in primary	179 (29)	47 (27)	1.5	0.9–2.6		
Completed primary	151 (24)	36 (24)	1.3	0.8–2.3		
Stopped in secondary	95 (15)	17 (18)	0.9	0.5–1.8		
Secondary 4 or above	142 (23)	27 (20)	1.0			
*p-value for trend test*			*0.03*			
Household wealth index						
1^st ^quintile (Poorest)	139 (20)	38 (29)	2.1	1.2–3.7**		
2^nd ^quintile	140 (20)	36 (26)	1.7	0.9–2.9		
3^rd ^quintile	150 (21)	33 (24)	1.3	0.8–2.5		
4^th ^quintile	140 (20)	36 (26)	1.1	0.6–2.0		
5^th ^quintile (Least poor)	140 (20)	23 (17)	1.0			
*p-value for trend test*			*0.01*			
Land ownership						
None	73 (10)	16 (22)	0.9	0.5 – 1.8		
1 acre or less	179 (25)	39 (23)	0.9	0.6 – 1.6		
2 acres	167 (24)	44 (28)	1.2	0.7 – 2.1		
3 or 4 acres	152 (22)	37 (25)	1.0	0.6 – 1.8		
5 acres or more	135 (19)	32 (24)	1.0			
*p-value for trend test*			*0.78*			
Hosmer-Lemeshow goodness-of-fit						
chi-square p-value					0.90	

*p < 0.05; **p < 0.01; ***p < 0.001

^a^Totals for some categories do not add up to 720 because of missing values

^b^Below -2 SD height-for-age of the NCHS/WHO reference

### Stunting distribution by socio-economic status

Unadjusted logistic regression analysis indicated that 3 socio-economic indicators (fathers' education, mothers' education and household wealth index) had a graded pattern with stunting across strata with the least educated or most poor being more likely to have stunted children. The corresponding p-values for test of trend was <0.001, 0.03 and 0.01 for mothers' education, fathers' education and household wealth index, respectively (Table 1). Land ownership however, showed no differentials with stunting across strata.

The extent of association between socio-economic indicators was examined. The correlations (Spearman's rho) between mothers' and fathers' education was 0.50; mothers' education and household wealth index = 0.37; mothers' education and land ownership = 0.12; fathers' education and household wealth index = 0.39; fathers' education and land ownership = 0.14; and household wealth index and land ownership = 0.18. Their colinearity was sufficiently low to allow them into the same model.

The four socio-economic indicators (mothers' education, fathers' education, household wealth index and land ownership), child age and sex were simultaneously entered in a backward conditional logistic regression. Only mothers' education, child age and sex appeared in the last step model. Children belonging to mother with no formal education or to mothers who stopped in primary school were significantly more likely to be stunted compared to their counterparts with mothers who were educated beyond primary school (OR = 2.1, Table 1). Being a boy (OR = 2.0) or older in age was also significantly associated with stunting.

### Stunting distribution by sex and socio-economic status

Stunting differentials for both sexes within and across socio-economic indicators did not favour male children. There was a graded relationship of stunting among males unlike females with almost all socio-economic groups except land ownership (Table 2). More males were stunted in poorer socio-economic strata than their peers in better off strata. The trend was more marked with mothers' education (p < 0.001) and household wealth index (p = 0.01), less with fathers' education (p = 0.06) and none with land ownership (p = 0.64). The magnitude of the difference in stunting between males and females diminishes with improvement in socio-economic status. The socio-economically advantaged groups show no differences in stunting status between the two sexes. Compared to the proportion of stunting between boys and girls in the strata of mothers educated above primary, mothers with no formal education were significantly more likely to have stunted boys than girls (OR = 3.0).

**Table 2:** Comparison of stunting status by sex and socio-economic position with unadjusted odds ratios and 95% confidence intervals (CI) derived from bivariate logistic regression with girls in the comparison group *(coefficients are expressed as odds ratios, p-values for test of trend and Pearson chi-square are also indicated)*

	Stunted children		p-value	Unadjusted odds ratio	95%CI
				
	Males n(%)	Females n(%)			
Mother's education					
None	33 (43)	12 (17)	0.01^f^	3.00	1.05–8.59*
Stopped in primary	44 (34)	36 (26)	0.18	1.33	0.53–3.38
Completed primary	15 (23)	7 (12)	0.16	2.34	0.69–7.87
Above primary	11 (17)	12 (14)	0.65	1.0	
*p-value for test of trend*	<*0.001*	*0.14*		*0.11*	
Father's education					
None	12 (40)	5 (19)	0.09^f^	1.65	0.45–6.03
Stopped in primary	30 (35)	17 (20)	0.04^f^	1.21	0.46–3.20
Completed primary	24 (31)	12 (17)	0.05^f^	1.37	0.49–3.87
Stopped in secondary	9 (21)	8 (16)	0.59	0.77	0.23–2.63
Ordinary level or above	16 (25)	11 (15)	0.14	1.0	
*p-value for test of trend*	*0.06*	*0.39*		*0.37*	
Index of household wealth					
1^st ^quintile (Poorest)	27 (40)	17 (25)	0.05^f^	2.17	0.81–5.81
2^nd ^quintile	25 (34)	14 (21)	0.05^f^	2.44	0.88–6.73
3^rd ^quintile	21 (35)	11 (15)	0.01^f^	2.60	0.90–7.56
4^th ^quintile	20 (28)	10 (13)	0.02^f^	2.73	0.92–8.09
5^th ^quintile (Least poor)	11 (15)	15 (22)	0.34	1.0	
*p-value for test of trend*	*0.01*	*0.34*		*0.28*	
Land ownership					
None	9 (27)	7 (18)	0.42	0.77	0.23–2.61
1 acre	19 (25)	20 (22)	0.59	0.57	0.22–1.48
2 acres	28 (41)	16 (18)	0.01^f^	1.05	0.41–2.70
3 or 4 acres	26 (30)	11 (18)	0.07^f^	1.42	0.52–3.87
5 acres or more	20 (30)	12 (18)	0.08^f^	1.0	
*p-value for trend test*	*0.64*	*0.66*		*0.17*	

^f^Fishers exact test; *p < 0.05

## Discussion

This study examined how socio-economic indicators namely mothers' education, fathers' education, household asset index and land ownership relate with inequalities in child health and nutrition using growth stunting as the proxy for the inequalities. Findings showed that mothers' education was a robust predictor for inequalities of child health and nutrition. Secondly, boys were more affected by low socio-economic status than girls. Indirectly the study also indicates that it may be unsuitable to bunch together socio-economic indicators as they could have low covariance or could conceptually not be interchangeable and thus unable to serve as adequate proxies for one another.

While interpreting findings of this study one should consider the fact that some of the fixed factors known to influence linear growth such as birth weight [7,8] and maternal stature [9,31] were not controlled for in the analysis. Birth weight was dropped because of many missing data attributable to the fact that majority of mothers in Uganda give birth outside the health units, consequently missing information on birth weight. Unfortunately the study design excluded assessment of maternal stature. Additionally, in a cross-sectional study such as the present one, it is impossible to say anything about cause and effect relationships. Nonetheless, considering that our agenda was to examine socio-economic predictors for inequalities in health and nutrition, these limitations do not greatly trivialise the importance of the study.

The socio-economic predictor variables used in the study were assumed to represent distinct dimensions reflecting different domains of influences from the society. The relatively low colinearity between the indicators used supports this assumption. This favours discussions on choice of socio-economic indicators where researchers are urged to use a number of socio-economic indicators rather bunching them together as each could have its unique contribution [20,32].

Our results regarding the relationship of socio-economic status with stunting are similar to findings in other studies [14-18]. As one climbs up the socio-economic ladder, there is a remarkable drop in the rate of stunting observed. Interestingly despite taking care of some covariance that existed between different socio-economic indicators during the analysis, mothers' education emerged as the only independent socio-economic predictor for inequalities of child health and nutrition. This indicates that the association of fathers' education and household wealth index with stunting could be confounded by mothers' education.

This emphasizes the need for promoting the education of the girl child with a principal aim of improving child health, which is in line with recommendations by UNICEF [33]. However, for this to be achieved there is need for a rational approach that involves a greater strategic and collaborative relationship between different sectors of government and agencies.

Additionally, mothers' education could be used as a suitable indicator to guide effective targeting especially with regard to identifying the most vulnerable families in rural subsistence agricultural communities. This is in appreciation of the fact that achieving universal coverage for child-health interventions presently lies far beyond the capacity of many health systems in low-income countries [34,35]. It is also consistent with the recommendation that modern programming has to go beyond equitable targeting [2] in order hasten improvements in health outcomes.

It was surprising that land ownership and access, perceived by many in Uganda as the ultimate form of security or socio-economic status [29], showed no differentials with stunting. Obviously, this may be different in more densely populated parts of the globe. The fact that majority (90%) of the households had a piece of land could also reduce differences in nutritional status among children. However, assuming no extraneous confounding or misclassification biases these findings could imply that land ownership in rural subsistence agricultural set-ups is not critical for child health and nutrition or is at least not as vital as parents' education and household wealth index in child well being.

Concordant with findings in other studies [31,36], age correlated positively with stunting. However, disturbing results were observed with child sex. Among well-nourished children, sex differences are attributed to a normal pattern of dimorphism, with males tending to be taller and heavier than females. Finding of this study indicate that male children were more likely to be stunted than females. Surprisingly several studies report a similar pattern in Africa [8,16,23,37]. What was more disturbing however was that larger proportions of male children were stunted as one descends the socio-economic profile as compared to females. Stunting differentials with socio-economic status observed in this study could be solely attributed to boys. Unfortunately none of the literature cited disaggregate stunting with sex across socio-economic groups. There are also no documented beliefs, attitudes or practices that segregate against the boy child in Uganda. However, in evolutionary biology there is evidence of male vulnerability in response to environmental stress in early life [38].

## Conclusions

This study highlights the importance of maternal education in child well being. From our data any increment in maternal education is likely to have a positive influence on child growth. Governments of low-income countries need to ensure that the girl child receives appropriate formal education. For effective targeting of families with children in greatest need or at highest risk of health and nutrition hazards, policy makers and programme managers could be guided by mothers' education. Findings that males appear to be more adversely affected by poverty than their female counterparts corroborate evidence from previous research.

## Authors' contributions

All authors participated in the design of the study, the interpretation of findings and write-up of the manuscript. HW coordinated and supervised field data collection and performed the statistical analysis. All authors read and approved the manuscript.

## Competing interests

The authors declare that they have no competing interests.
